# Examining the quality and sample representativeness of linked 1958 National Child Development Study and Hospital Episode Statistics data.

**DOI:** 10.23889/ijpds.v7i3.1990

**Published:** 2022-08-25

**Authors:** Richard Silverwood, Nasir Rajah, Lisa Calderwood, Bianca De Stavola, Katie Harron, George Ploubidis

**Affiliations:** 1 Centre for Longitudinal Studies, UCL Social Research Institute, University College London; 2 Population, Policy & Practice Department, UCL Great Ormond Street Institute of Child Health, University College London

## Objectives

It is important to evaluate the quality of linkages between cohort and administrative data to discern the likely reliability of research using the linked data resource. We examined the quality and sample representativeness of linked 1958 National Child Development Study (NCDS) and Hospital Episode Statistics (HES) data.

## Approach

We examined the quality of the linkage in terms of the associations between key cohort member sociodemographic characteristics and successful linkage, and compared the levels of successful linkage within strata of NCDS variables which may be expected to be associated with hospital attendance (self-reported hospital attendance, self-rated general health, self-reported long-term illness), and hence with successful HES linkage. We additionally evaluated the population representativeness of the linked sample using external population statistics (hospital admission rates in the general population).

## Results

Some sociodemographic characteristics were found to be associated with successful HES linkage, with females slightly more likely than males (risk ratio 1.03; 95% confidence interval 1.00, 1.06) and those who had ≤ 1 person per room at birth more likely than those who had > 1.5 (1.12; 1.05, 1.19) to have linked data. There was a high level of correspondence between NCDS variables and HES linkage – e.g., 86.0% of cohort members who reported no day patient or in-patient attendance had no linked HES Admitted Patient Care (APC) data and 76.3% of those who reported having day patient or in-patient attendance did have linked HES APC data. The level and pattern of hospital admission rates over time was similar in the linked NCDS-HES data to the population statistics.

## Conclusion

The quality and representativeness of the linked NCDS-HES data appears to be high. We hope that these analyses will both improve the quality and transparency of research using this linked data resource and encourage providers and users of other linked data resources to undertake and publish similarly thorough evaluations.

